# Ovarian Filariasis in a Wild Southern Tamandua (*Tamandua* *tetradactyla;* Mammalia: Myrmecophagidae)

**DOI:** 10.3390/pathogens11080918

**Published:** 2022-08-15

**Authors:** Lilja Fromme, Débora Regina Yogui, Mario Henrique Alves, Josué Díaz-Delgado, Arnaud Leonard Jean Desbiez, André Luis Quagliatto Santos, Juliana Mariotti Guerra, Marion Langeheine, Ursula Siebert, Ralph Brehm, José Luiz Catão-Dias, Pedro Enrique Navas-Suárez

**Affiliations:** 1 Institute for Terrestrial and Aquatic Wildlife Research, University of Veterinary Medicine Hannover, 30173 Hannover, Germany; 2 Institute for Anatomy, University of Veterinary Medicine Hannover, 30173 Hannover, Germany; 3 Institute for Conservation of Wild Animals (ICAS), Campo Grande 79070-180, Brazil; 4 Nashville Zoo, Nashville, TN 37211, USA; 5 Postgraduate Program in Ecology and Conservation (PPGEC), Federal University of Mato Grosso do Sul, Campo Grande 79070-900, Brazil; 6 Laboratory of Wildlife Comparative Pathology (LAPCOM), School of Veterinary Medicine and Animal Science, University of São Paulo, São Paulo 05508-270, Brazil; 7 Royal Zoological Society of Scotland (RZSS), Murrayfield, Edinburgh EH12 6TS, UK; 8 Laboratory of Wild Animal Research (LAPAS), Faculty of Veterinary Medicine, Federal University of Uberlândia, Uberlândia 38405-302, Brazil; 9 Center of Pathology, Adolfo Lutz Institute, São Paulo 01246-902, Brazil

**Keywords:** Xenarthra, anteater, Mammalia, Filarioidea, parasite, reproductive health

## Abstract

Knowledge of reproductive health in wild southern tamanduas (*Tamandua tetradactyla*; Mammalia: Myrmecophagidae) is fragmentary. During necropsies of roadkill xenarthran species in Brazil, a case of ovarian filariasis in an adult female southern tamandua was observed. Macroscopically, both ovaries were irregularly enlarged and had numerous smooth protuberances. Histologically, the affected ovarian parenchyma presented adult nematodes (including females with microfilaria) surrounded by pleocellular inflammatory infiltrates. The morphological characteristics of the nematodes were consistent with the superfamily Filarioidea (order Spirurida). The adjacent ovarian parenchyma had developing and atretic follicles at different stages of maturation. Filarial nematodes were not observed in other tissues. The cause of death of this tamandua was fatal acute polytrauma as a consequence of the motor vehicle collision. This case adds to a prior report of ovarian filariasis in two southern tamanduas in Nicaragua and Guatemala, dating back almost 100 years, and suggests filarial infections could potentially have an impact on reproductive success in southern tamanduas and possibly other xenarthrans. Several xenarthran species are under different levels of threat and knowledge of their basic reproductive health is crucial for conservation programs.

## 1. Introduction

The southern tamandua (*Tamandua tetradactyla*, Linnaeus, 1758) belongs to the suborder Vermilingua (order Pilosa) which comprises three genera of anteaters: *Tamandua*, *Myrmecophaga* and *Cyclopes* [1]. Together with sloths and armadillos, anteaters form the superorder Xenarthra and occupy a basal position in the placental mammal tree [2,3,4]. Xenarthra share some peculiar skeletal and physiological characteristics, such as additional vertebral articulations [1] and a low body temperature [5]. Furthermore, some particular reproductive features have been described, as intraabdominal testes in male Xenarthra [6,7], a simple uterus and the absence of a cervix in female specimens [8,9].

Despite their distinguished phylogenetic position among placental mammals and their peculiar characteristics, Xenarthra have been scarcely studied [10] and knowledge on reproductive health of the southern tamandua is fragmentary [11,12]. A few cases of pathological conditions of the reproductive organs in female captive and wild anteaters were reported in Brazil: endometritis in giant anteaters (*Myrmecophaga tridactyla*) and southern tamanduas [13,14], and ovarian hypoplasia and paraovarian cysts [13]. Furthermore, ovarian filarial nematodes were described in two wild southern tamanduas from Guatemala and Nicaragua in a report dating back almost 100 years [15]. Here, we present pathological details of another rare case of ovarian filariasis in an adult wild southern tamandua from Brazil.

## 2. Material and Methods

### 2.1. Sample Collection and Tissue Processing

Over a three-year period (February 2017 to 2020), a systematic monitoring of wild xenarthran species killed by motor vehicle collisions was carried out in Mato Grosso do Sul (MS) State, Brazil. In the course of the project, an adult female southern tamandua with ovarian filariasis was necropsied and gross post-mortem examination including a detailed evaluation of reproductive organs along with photo documentation was conducted. Tissue samples (tongue, lungs, stomach, small intestine, spleen, adrenal glands and reproductive organs) were collected and processed for histopathological analysis following standard protocols [16]. Macro and microscopic findings in reproductive organs were compared to the normal reproductive organs of another three adult female southern tamanduas necropsied in the same project.

### 2.2. DNA Extraction and PCR

In an attempt to obtain genetic material for molecular identification of the species of filaria observed microscopically in the ovaries of the southern tamandua, four sections of 9 µm of the formalin-fixed and paraffin-embedded ovarian tissue were applied for DNA extraction through the ReliaPrep™ FFPE gDNA Miniprep System (Promega Corporation, Madison, MI, USA), according to manufacturer’s instructions. To evaluate the quality of the genetic material, a polymerase chain reaction (PCR) to amplify pan-eukaryotic 18S rRNA gene was performed with 18S-252F (5′-CAG CCA CCC GAG ATT GAG CA-3′) and 18S-252R (5′-TAG TAG CGA CGG GCG GTG TG-3′) primers and GoTaq^®^ Hot Start Green Master Mix (Promega Corporation, Madison, WI, USA). Thermal cycler conditions were 5 min at 95 °C; 40 cycles of 95 °C for 30 s, 55 °C for 60 s, and 72 °C for 30 s; and a final extension step at 72 °C for 10 min. Nuclease-free water was used as negative control in all PCR and reverse transcription quantitative (RT-q)PCR assays. PCR products were separated by electrophoresis in 2% agarose gels, stained with GelRed (Biotium, Fremont, CA, USA), and examined under ultraviolet light.

The study was performed under national licence (#53798-10) by the Biodiversity Authorization and Information System (58745-1 SISBIO, Brazil) and approved by the FMVZ/USP Ethics Committee on Animal Use in Research (#7198020317). 

## 3. Results

The necropsied female southern tamandua with a body weight of 5 kg was in good body condition and classified as adult [5,17]. The main gross pathological findings were related to injuries caused by the motor vehicle collision: rupture of internal organs (brain, lungs, liver, stomach, small intestine, kidneys), muscular rupture (abdominal, diaphragm), haemothorax, splenic haematoma, subcutaneous abdominal haematoma, bone fractures (nasal, occipital, parietal, temporal, left humerus), haemorrhage of ear and nostrils, and multifocal cutaneous abrasions and lacerations (forelegs, hindlimbs, head and neck). 

**Figure 1:**
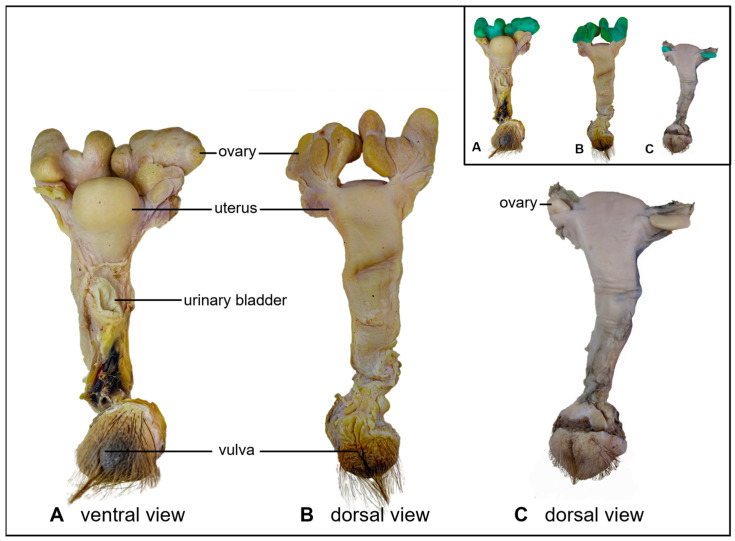
Macroscopic aspects of the reproductive organs of a female southern tamandua with ovarian filariasis in comparison to reproductive organs of an age range-matched control specimen. (**A**,**B**) ventral and dorsal view of the reproductive organs of the specimen with ovarian filariasis, (**C**) dorsal view of the reproductive organs of an unaffected specimen. Inset: ovaries are highlighted in green.

**Figure 2:**
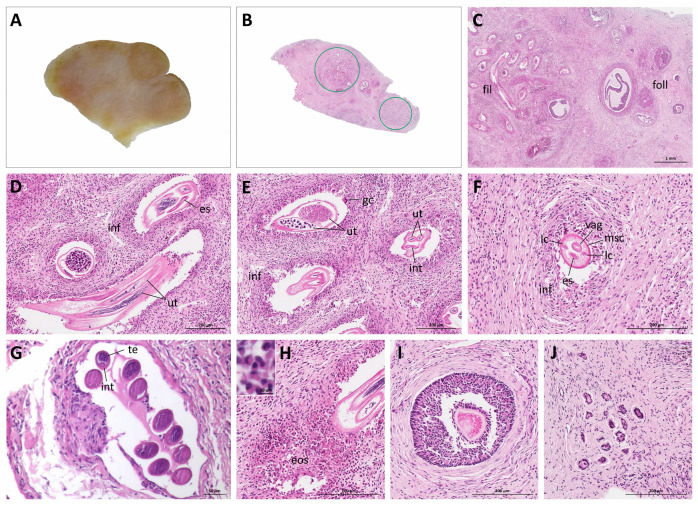
Macroscopic and microscopic findings in the ovary of a southern tamandua with ovarian filariasis. (**A**) Gross and (**B**) microscopic features (longitudinal section through the ovary); encircled: sections of filarial nematodes in ovarian tissue. (**C**) Ovarian tissue with sections of filarial nematodes next to ovarian follicles; fil: filaria; foll: follicles, including degenerating tertiary follicles. (**D**–**F**) Sections through female filaria in the ovarian tissue; ut: double uterus; vag: vagina; es: esophagus; int: intestine; msc: coelomyarian musculature; lc: lateral chords; inf: inflammatory infiltrate; gc: multinucleated giant cell. (**G**) Sections through male filaria in the ovarian tissue; te: testis; int: intestine. (**H**) Eosinophilic infiltrates; eos: eosinophils; inset: higher magnification. (**I**,**J**) Ovarian follicles, (**I**) late secondary follicle, (**J**) primordial follicles. HE stain.

The reproductive organs were not affected by trauma, but on gross inspection both ovaries were irregularly enlarged with numerous smooth protuberances (Figure 1). The left ovary was 28 mm (length) × 20 mm (width) × 4 mm (depth); the right ovary was 27 mm × 19 mm × 7 mm. According to Rossi et al. [9], the normal ovaries in the southern tamandua are ovoid and the left ovary measures 17.27 ± 7.2 mm × 7.04 ± 2.57 mm × 5.46 ± 2.58 mm and the right ovary 15.69 ± 5.18 mm × 6.43 ± 1.31 mm × 4.98 ± 1.18 mm. The three unaffected control specimen necropsied during this study also showed ovoid ovaries and ovarian measurements, taken only from one specimen, fitted well in the scope of measurements reported by Rossi et al. [9]: 18 mm × 5.5 mm × 4 mm (left ovary) and 21 mm × 10 mm × 6 mm (right ovary). Upon longitudinal section, the ovarian parenchyma of the affected specimen was grey-white and had multifocal to coalescent, poorly defined, yellow nodules primarily within the cortical region (Figure 2A). Only sporadically, cut follicles were observed. In comparison, the ovarian parenchyma of the unaffected specimens was uniformly grey-white with numerous cut follicles or corpora lutea.

Histologically, the ovarian nodules aforementioned consisted of multiple sections of adult nematodes, surrounded by moderate pleocellular eosinophilic and granulomatous inflammatory infiltrates with multinucleated giant cells (foreign body type) and granulation tissue (Figure 2B–H). Cross-sections of adult nematodes were characterized by a thin cuticle, coelomyarian musculature, lateral chords and a pseudocoelom with a small digestive tube and a double uterus with microfilaria in females, and a single testis containing sperm in males (Figure 2D–G). These histomorphological features were consistent with the superfamily Filarioidea (order Spirurida) [18,19]. The non-affected adjacent ovarian parenchyma had follicles at different developmental stages, ranging from primordial to atretic tertiary follicles. In the sections examined, no corpora lutea or albicantia were observed.

Unfortunately, due to DNA fragmentation, a molecular characterization of the filarial nematode could not be performed. In a PCR for amplifying the pan-eukaryotic 18S rRNA gene, amplification was negative and only a diffuse smear was visualized in the electrophoresis. 

Filarial nematodes were not observed in other tissues of the female southern tamandua. Microscopic findings of other organs included an intrasarcolemal protozoal cyst consistent with *Sarcocystis* sp. in the tongue without associated inflammation; moderate multifocal subpleural and alveolar oedema with haemorrhage; mild to moderate multifocal granulomatous gastritis; mild to moderate multifocal eosinophilic enteritis; mild to moderate white pulp hyperplasia and mild multifocal haemosiderosis in the spleen and mild to moderate diffuse cortical (fascicular-reticular) loss and fibrosis in the adrenal glands. The cause of death of the female southern tamandua was fatal acute polytrauma as a consequence of the motor vehicle collision. Bilateral nematodal oophoritis was considered an incidental finding. Filarial nematodes were not observed in ovaries of the three control specimens on histological examination. 

## 4. Discussion and Conclusions

Ovarian parasitic disease is rare, both in women [20,21], and domestic and wild mammalian species [22]. In women, the most common causes of helminth-induced oophoritis are schistosomiasis, enterobiasis, and echinococcosis [23,24,25,26]. Moreover, a few studies in women have reported filarial nematodes (e.g., *Wuchereria bancrofti, Brugia* spp.) in ovaries, mostly in dilated lymphatic vessels [27,28,29,30,31]. In domestic and wild mammalian species, there are only few reports of parasitic oophoritis caused by aberrant helminth migration, including e.g., mesocestoides and schistosoma [32,33]. To the best of our knowledge, there is only one single previous record of ovarian filariasis in mammalian wild species documenting ovarian infestation with filarial nematodes in two southern tamanduas from Nicaragua and Guatemala [15]. Gross and microscopic findings similar to the characteristics described in the present report, were observed in these tamanduas: grossly, the ovaries were irregularly enlarged and microscopically, filarian nematodes were observed in lymphatic vessels and the ovarian stroma, surrounded by eosinophilic infiltrates as well as granulation tissue [15]. By contrast, we did not detect filarid lymphovascular involvement in the present tamandua. Furthermore, Wislocki [15] described ovarian follicles from primary to tertiary (Graafian) interspersed between parasitic stages and no other tissues showed signs of nematodiasis. The genus and species of the filarial parasite could not be determined, neither in the present study nor by Wislocki [15], on the basis of histologic sections. 

There is a general lack of data on the occurrence of filarids in anteaters. Only one species, *Chabfilaria freitaslenti*, has been described in the giant anteater [34,35]. Furthermore, unidentified microfilariae were seen in a blood smear from a free-ranging southern tamandua [13]. Filarids reported in other xenarthran species include *Orihelia anticlava*, *Dirofilaria* *immitis*, *Acanthocheilonema sabanicolae, Strianema venezuelensis*, and *Dipetalonema* *(Dasypafilaria) averyi* in armadillos [36,37,38,39,40,41] and *Dirofilaria* spp. and *Chabfilaria jonathani* in sloths [34,42]. All filarial species described in Xenarthra, so far, belong to the family Onchocercidae. 

Filarial nematodes are typically found in tissue or within lymphovascular structures. Their life cycle depends on insect vectors who ingest filarial eggs or first-stage larvae (microfilariae) by feeding on tissue fluids or blood of host species. The eggs or microfilaria then develop into infectious larvae and are transmitted when the vector feeds on the final host where larvae develop into adult stages [43]. 

Reports on reproductive pathology in the female southern tamandua are scarce and only a small number of specimens has been examined (likewise, the present study comprises only 4 female specimens). In this light, the number of three reported cases of ovarian filariasis from different geographical regions (the present study and two cases reported by Wislocki [15]) raises the question whether the species is particularly susceptible to this form of parasitism. There might be an impact on reproductive success of affected specimens as the inflammatory process in the ovary replaced areas of follicular development and enlargement of the ovaries could impede the capture of ovulated oocytes by the ampulla of the uterine tube.

Future studies may investigate the prevalence of ovarian filariasis in wild southern tamanduas as well as other xenarthrans. Several xenarthran species are classified as Vulnerable or Near Threatened by the International Union for Conservation of Nature [44] and knowledge of their reproductive health is crucial for conservation programs and wildlife health surveillance.

## Data Availability

The complete dataset used and analyzed during the current study are available from the corresponding author on reasonable request.
